# Multifunctional Biocomposites: Synthesis, Characterization, and Prospects for Regenerative Medicine and Controlled Drug Delivery

**DOI:** 10.3390/molecules29153483

**Published:** 2024-07-25

**Authors:** Mohamed Aaddouz, Ridouan El Yousfi, Rachid Sabbahi, Khalil Azzaoui, Meryem Idrissi Yahyaoui, Abdeslam Asehraou, Belkheir Hammouti, Fouad Laoutid, Mohammed M. Alanazi, Elmiloud Mejdoubi

**Affiliations:** 1 Laboratory of Applied Chemistry and Environment, Department of Chemistry, Faculty of Sciences. Mohammed First University, Oujda 60000, Morocco; aaddouzmed@gmail.com (M.A.); red1elyousfi@gmail.com (R.E.Y.); ee.mejdoubi@gmail.com (E.M.); 2 Research Team in Science and Technology, Higher School of Technology, Ibn Zohr University, Quartier 25 Mars, Laayoune 70000, Morocco; 3 Euromed Research Center, Euromed Polytechnic School, Euromed University of Fes, UEMF, Fes 30030, Morocco; k.azzaoui@yahoo.com (K.A.); hammoutib@gmail.com (B.H.); 4 Engineering Laboratory of Organometallic, Molecular Materials and Environment, Faculty of Sciences, Sidi Mohammed Ben Abdellah University, Fez 30000, Morocco; 5 Laboratory of Bioresources, Biotechnology, Ethnopharmacology and Health, Faculty of Sciences, Mohammed First University, Oujda 60000, Morocco; iy.meryem@ump.ac.ma (M.I.Y.); asehraou@yahoo.fr (A.A.); 6 Laboratory of Polymeric & Composite Materials, Materia Nova Research Center, 3 Avenue Nicolas Copernic, B-7000 Mons, Belgium; fouad.laoutid@materianova.be; 7 Department of Pharmaceutical Chemistry, College of Pharmacy, King Saud University, Riyadh 11451, Saudi Arabia; mmalanazi@ksu.edu.sa

**Keywords:** antimicrobial, biomaterials, co-precipitation, drugs, hydroxyapatite, multifunctional

## Abstract

This article presents a new method for preparing multifunctional composite biomaterials with applications in advanced biomedical fields. The biomaterials consist of dicalcium phosphate (DCPD) and bioactive silicate glasses (SiO_2_/Na_2_O and SiO_2_/K_2_O), containing the antibiotic streptomycin sulfate. Materials were deeply characterized by X-ray diffraction and attenuated total reflectance Fourier transform infrared spectroscopy, and zeta potential analysis, UV–visible spectrophotometry, and ion-exchange measurement were applied in a simulating body fluid (SBF) solution. The main results include an in situ chemical transformation of dicalcium phosphate into an apatitic phase under the influence of silicate solutions and the incorporation of the antibiotic. The zeta potential showed a decrease in surface charge from ζ = −24.6 mV to ζ = −16.5 mV. In addition, a controlled and prolonged release of antibiotics was observed over a period of 37 days, with a released concentration of up to 755 ppm. Toxicity tests in mice demonstrated good tolerance of the biomaterials, with no significant adverse effects. Moreover, these biomaterials have shown potent antibacterial activity against various bacterial strains, including *Listeria monocytogenes*, *Staphylococcus aureus*, *Escherichia coli*, and *Pseudomonas aeruginosa*, suggesting their potential use in tissue engineering, drug delivery, and orthopedic and dental implants. By integrating the antibiotic into the biomaterial composites, we achieved controlled release and prolonged antibacterial efficacy. This research contributes to advancing biomaterials by exploring innovative synthetic routes and showcasing their promise in regenerative medicine and controlled drug delivery.

## 1. Introduction

The restoration of bone defects has been a central concern in regenerative medicine, captivating the attention of many researchers for years. Various studies have been carried out to develop biomaterials for clinical applications [1,2]. These biomaterials must be bioactive and composed of polymers, whether synthetic or natural, ceramics, or metals [3,4].

Among them, hydroxyapatite (HAp), a calcium phosphate, is widely recognized for its excellent compatibility with bone tissue due to its similarity to natural bone minerals [5,6]. 

HAp is valued for its ability to adjust its crystallinity and microstructure to meet specific needs, thanks to its biodegradable properties and biocompatibility, ensuring that it is non-toxic, non-inflammatory, and enhances the immune response [5,7]. However, HAp can present certain limitations, particularly in terms of mechanical and chemical properties. Traditional synthesis methods require extremely high purity [8,9], which can result in the loss of crucial elements such as sodium, silicon, and potassium, essential for bone growth and biological functions [10,11,12]. In contrast, brushite (dicalcium phosphate dihydrate, DCPD) is metastable under physiological conditions and resorbs faster than apatitic cements, while having the ability to transform into hydroxyapatite in vivo [13,14].

This study proposes a new approach by developing “HAp/bio-glass” biocomposites by precipitating HAp in silicate solutions based on the hydrolysis of dicalcium phosphate dihydrate. This process aims to strengthen interactions between the final phases of composite biomaterials, create a favorable chemical environment for the deposition of apatitic particles, and promote synergy between biocomposite components. This method also enables the material to be consolidated at low temperatures, facilitating the integration of active ingredients, unlike conventional bioceramics which require sintering at very high temperatures [15,16,17].

Research on sustained-release delivery systems plays a key role in the mitigation of antibiotic resistance. In the context of anti-infective treatments, which often require prolonged treatment durations, adequate exposure to antibiotics is essential for the eradication of microorganisms [18]. However, patient compliance remains a challenge. Patients often discontinue treatment once they feel better, contributing inadvertently to incomplete antibiotic treatment [19]. This incomplete treatment can exacerbate the development of antibiotic resistance. In addition, many antibiotics have a short half-life, requiring frequent administration. To solve these problems, adaptable drug delivery systems have emerged [18,19]. These innovative systems not only overcome biological barriers, but also ensure precise, prolonged and controlled drug release at the target site. Furthermore, they have the ability to incorporate multiple drugs in the same delivery vehicle.

One of the greatest challenges facing contemporary medicine in the treatment of chronic diseases such as osteomyelitis and osteoporosis is antibiotic resistance and the control of drug release [20,21]. A major effort has been made by specialists to develop materials capable of releasing drugs precisely and with reproducible and predictable kinetics. Although most of these drug carriers are polymers, certain inorganic materials can also play an important role.

The aim of this integrated approach is to deepen our understanding of the properties and performance of multifunctional biocomposites, with a view to exploring their potential applications in medicine. Various strategies have been investigated for combining antibiotics with phosphocalcic materials to extend the release period, with the aim of optimizing integration methods to regulate the release rate. This study will address several key steps, from the synthesis of the composite biomaterial to the analysis of its drug release, as well as structural and surface characterization, and will conclude with the exploration of its antibacterial efficacy, thus fostering a deeper understanding of the multifunctional biocomposite and its potential biomedical applications.

## 2. Results and Discussion

### 2.1. Characterization of Multifunctional Composite Biomaterials

#### 2.1.1. Characterization by X-ray Diffraction

**Figure 1:**
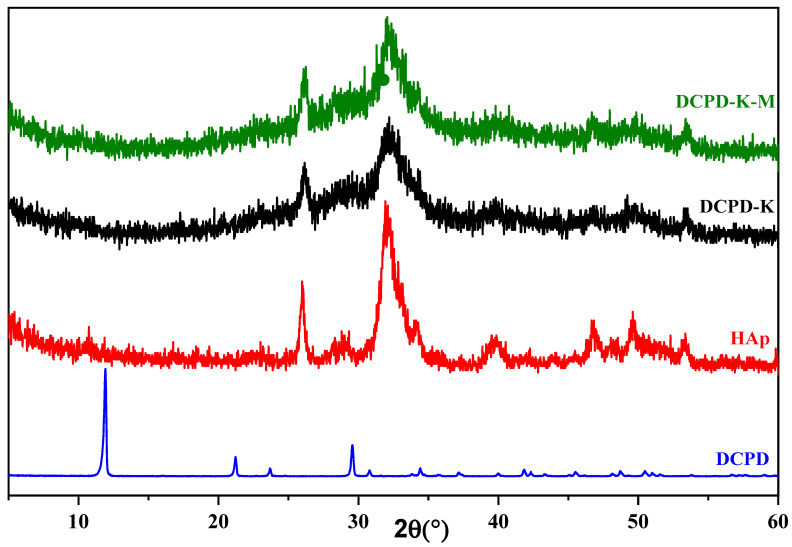
Diffractograms of DCPD-K and DCPD-K-M composite biomaterials, HAp, and DCPD.

**Figure 2:**
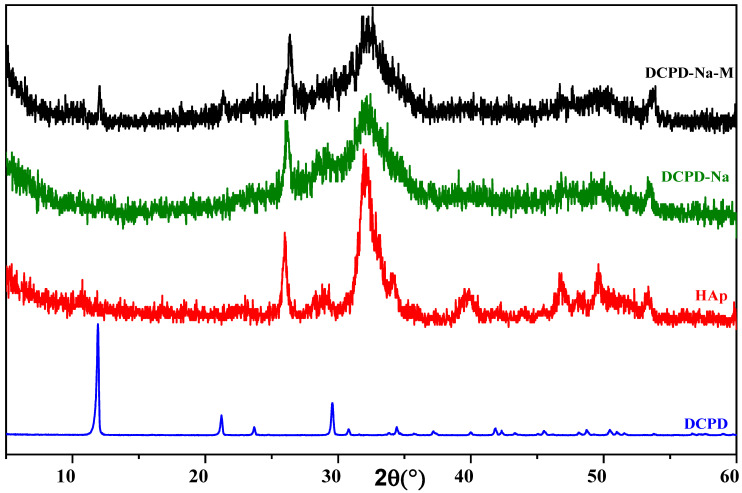
Diffractograms of DCPD-Na and DCPD-Na-M composite biomaterials, HAp, and DCPD.

X-ray diffraction characterization of the engineered biomaterials was carried out on DCPD samples synthesized using the double decomposition method, which had previously been chosen at the start of this study, in the presence of bio-glasses (SiO_2_/Na_2_O and SiO_2_/K_2_O), followed by consolidation. The X-ray diffraction pattern is shown in Figure 1 and Figure 2, where it can be seen that in both the SiO_2_/Na_2_O and SiO_2_/K_2_O bio-glass cases, dicalcium phosphate evolves in situ towards a poorly crystallized apatitic phase.

Combining the XRD diagrams of the composite biomaterials after the drug has been introduced into the different silicate solutions, for the DCPD/SiO_2_-Na_2_O and DCPD/SiO_2_-Na_2_O/cured antibiotic composite biomaterials (Figure 1), we see an in situ chemical transformation towards the apatitic phase. The diffractograms reveal broad peaks at 2θ = 26.42° and 2θ = 32°, characteristic of the apatitic phase, in both cases, whether in the absence of antibiotic (DCPD-K) or in its presence (DCPD-K-M). For the cured DCPD-Na and DCPD-Na-M biocomposites (Figure 2), their XRD spectra clearly demonstrate in situ chemical transformation towards a poorly crystallized apatitic phase.

The results of X-ray diffraction analysis highlight the importance of the chemical conditions imposed by silicate media, in particular pH conditions, on the nature of composite biomaterials. Similarly, the nature of the cations (Na+ and K+) plays a significant role in the in situ chemical evolution of dicalcium phosphate towards the apatitic phase in the presence or absence of the antibiotic.

#### 2.1.2. Characterization by Infrared Spectroscopy

**Figure 3:**
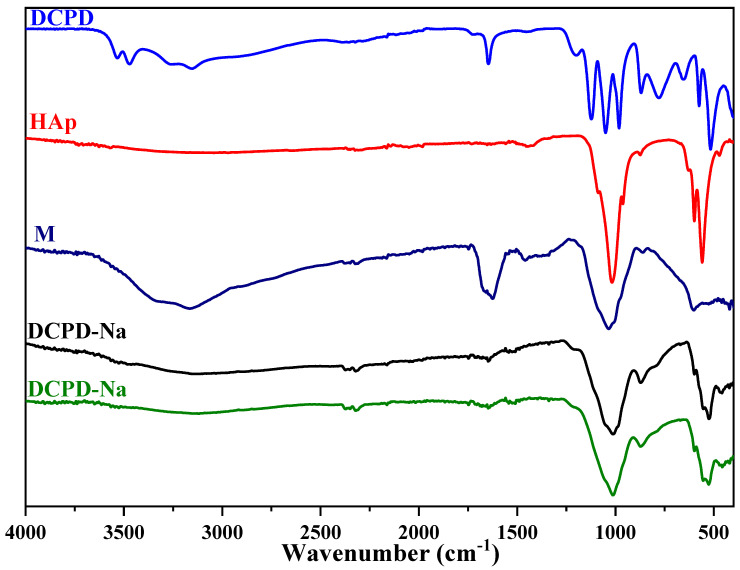
IR spectra of DCPD-Na and DCPD-Na-M composites, antibiotic M, HAp, and DCPD.

**Figure 4:**
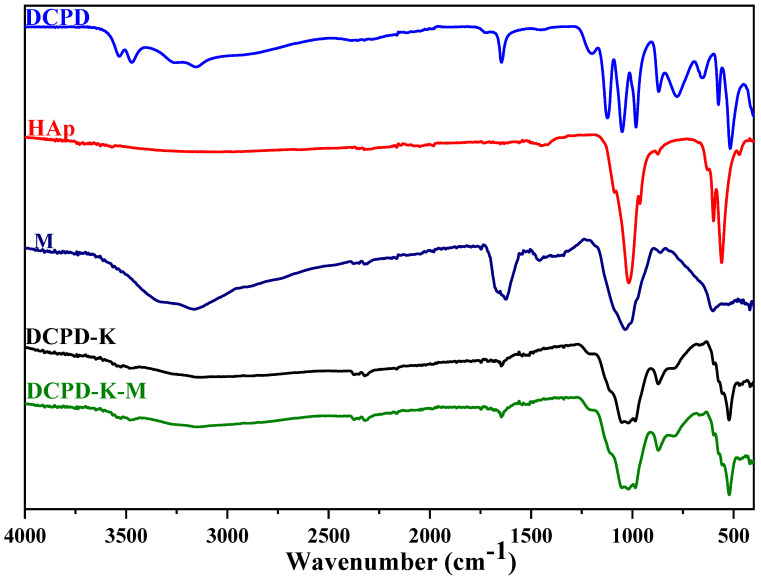
IR spectra of DCPD-K and DCPD-K-M composites, antibiotic M, HAp, and DCPD.

In this study, we explored the characteristics of HAp/bio-glass composite biomaterials, incorporating the antibiotic streptomycin sulfate, using infrared spectroscopy analysis (Figure 3 and Figure 4). Our main objective was to understand how the various dicalcium phosphate groups evolve in situ in the presence of various silicate solutions, and the impact of the presence or absence of antibiotics.

The infrared absorption spectra depicted in Figure 3 and Figure 4 illustrate the in situ evolution of brushite towards an apatitic phase. This transformation occurs in the absence of antibiotics but in the presence of various silicate solutions. Analyzing these FTIR spectra reveals several key features: First, the characteristic DCPD bands between ν = 550 cm^−1^ and ν = 1500 cm^−1^ vanish. Second, the band width increases between ν = 1200 cm^−1^ and ν = 700 cm^−1^. Third, HAp bands emerge [22]. These results clearly indicate an in situ chemical conversion of DCPD to an apatitic phase in the presence of SiO_2_/Na_2_O and SiO_2_/K_2_O bio-glasses. These findings align with previous observations from X-ray diffraction, confirming the formation of HAp/SiO_2_/Na_2_O and HAp/SiO_2_/K2O composite biomaterials.

With regard to the specific DCPD-Na composite biomaterial, whether or not associated with streptomycin sulfate (Figure 3 and Figure 4), several important observations were made: (i) the shift of the band at 3151 to 3143 cm^−1^, associated with elongations of the O-H bond in hydroxyapatite, suggests changes in the chemical environment of the hydroxyl groups [23], (ii) the presence of C-H group vibrations at 3100 cm^−1^ confirms the incorporation of these groups into the HAp/SiO_2_/Na_2_O structure, and (iii) the appearance of the band at 1679 cm^−1^, corresponding to the stretching vibrations of the amide group (−C=O−NH−) present in streptomycin sulfate, confirms the presence of this antibiotic in the composite biomaterial.

Other band shifts, notably at 1648, 1340, 1213, and 871 cm^−1^, as well as bands at 525, 553, and 597 cm^−1^, correspond to vibrations characteristic of the carbonate ions (CO_3_^2−^) [24,25] introduced into the apatite structure and the phosphate ions (PO_4_^3−^) of apatite [26].

These observations clearly demonstrate the complex interactions between bio-glass, apatite, and streptomycin sulfate, confirming changes in the chemical environment of these compounds and validating the formation of the HAp/SiO_2_/Na_2_O-M composite biomaterial.

The infrared spectra of the DCPD-K and DCPD-K-M composite biomaterials (Figure 3 and Figure 4) also reveal a chemical transformation of the DCPD phase in situ towards an apatitic phase in both cases. Furthermore, the observation of absorption bands between 1250 and 750 cm^−1^, as well as the appearance of bands associated with antibiotic and apatite in the interval between 3700 and 2500 cm^−1^, confirms the formation of the HAp/SiO_2_/K_2_O-M composite biomaterial, implying the combined presence of apatite, silicate, and antibiotic.

### 2.2. Study of Biomaterial Surface Charges

Zeta potential, an important measurement in materials science and surface chemistry, is used to assess the surface charge of composite biomaterials. In our study, the surfaces of the biomaterials are negatively charged (Table 1). We observed a significant difference between the zeta potential of the phosphate/bio-glass composite biomaterial and that of the two/bio-glass-M composite biomaterials.

In contrast, other analytical techniques, such as IR spectroscopy and X-ray diffraction (XRD) of DCPD-Na and DCPD-K composites, show an in situ evolution towards an apatite phase. This transition to an apatite phase leads to the formation of agglomerates, highlighted by zeta potential values. This conclusion is reinforced by our preceding results.

After insertion of the antibiotic, values of zeta potentials decreased in the case of DCPD-Na composite biomaterials (ζ = −23.9 mV) compared to the presence of the antibiotic (DCPD-Na-M, ζ = −19.7 mV). Similarly, for DCPD-K composite biomaterials, the values of zeta potentials decreased from ζ = −24.6 mV to ζ = −16.5 mV (DCPD-K-M). This could be attributed to interactions between the biomaterials and the antibiotic, as well as to the formation of particles with a greater tendency to agglomerate due to the presence of the antibiotic [27,28].

### 2.3. Antibiotic Release Into SBF Solution

**Figure 5:**
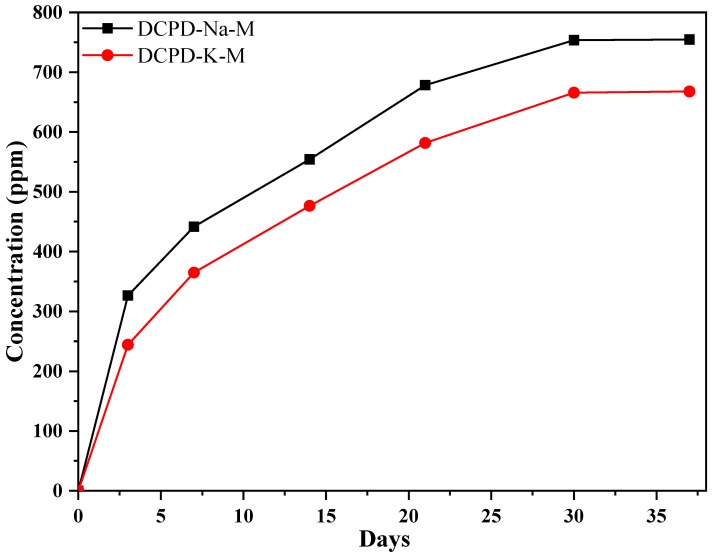
Kinetics of streptomycin sulfate release from the DCPD-Na-M and DCPD-K-M composite biomaterials.

To monitor the kinetics of antibiotic release from the composite (Figure 5), we used a UV–visible spectrophotometer. This method enabled us to assess the concentration of the antibiotic in the SBF solution. These measurements were obtained using a calibration curve correlating the absorbance at 203 nm, derived from the UV–visible spectrum, with the concentration of the antibiotic “streptomycin sulfate” in ppm.

It is noticeable that after three days of incubation at 37 °C, the DCPD-Na-M composite biomaterial (326 ppm) rapidly releases more antibiotic than the DCPD-K-M composite biomaterial (244 ppm). Both DCPD-Na-M and DCPD-K-M biocomposites have the advantage of trapping the antibiotic within the apatite formed in situ, which is characterized by the presence of tunnels. In addition, electrostatic interactions between the antibiotic and the apatite are formed, reinforced by the presence of silicate in the form of bio-glass surrounding the apatite.

The DCPD-K-M composite releases less antibiotic due to the presence of potassium silicate, compared to the DCPD-Na-M composite. This difference could be explained by the difference in ionic radii between potassium and sodium. On the other hand, the controlled long-term release may be attributed to in situ precipitation of hydroxyapatite in the presence of silicate solutions, which could further promote phosphate/bio-glass interactions and synergy between the constituents of the phosphate/bio-glass/antibiotic composite.

### 2.4. Ion Exchange with SBF Medium

**Figure 6:**
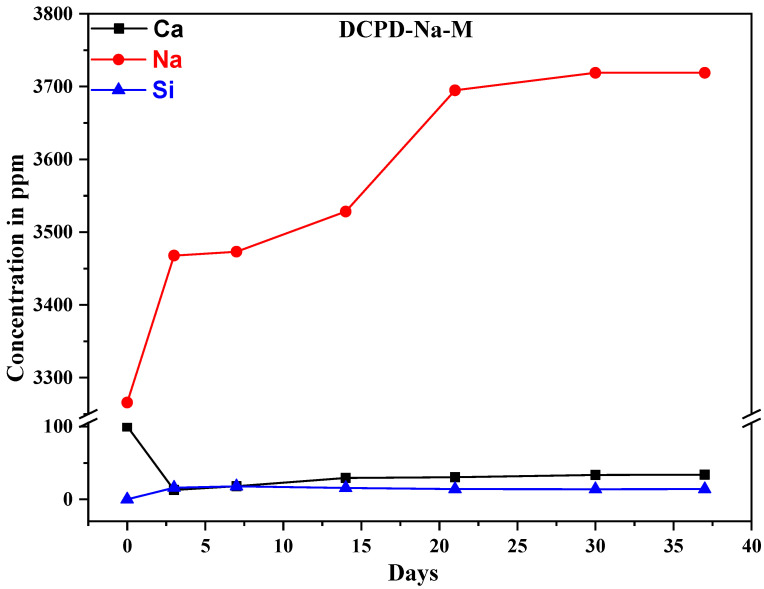
Concentrations of the elements Ca, Na, and Si released by the DCPD-Na-M composite biomaterial.

The use of ICP-AES enables highly sensitive measurements to be carried out, and precise data to be obtained (sensitivity of approximately 1 ppm, depending on the matrix analyzed). This technique can be used to determine the ion exchange between composite biomaterials and the SBF solution. The measurements were carried out on the SBF solution used during the immersions. The results show variations in the concentration of the following elements: silicon, calcium, potassium, and sodium (Figure 6). Silicon content is an indicator of bio-glass dissolution, while calcium content is an indicator of phosphocalcic exchange, leading to the formation of an apatitic layer favored by favorable pH conditions.

**Figure 7:**
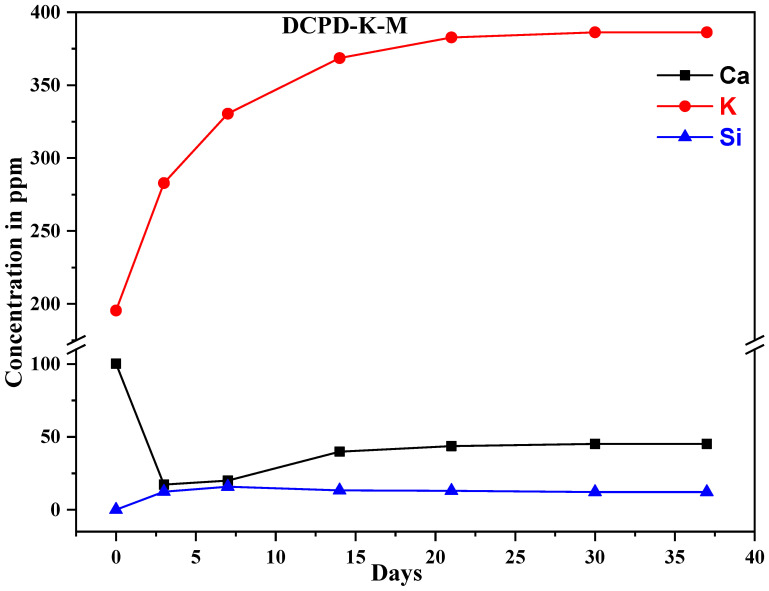
Concentrations of the elements Ca, K and Si released by the DCPD-K-M composite biomaterial.

In addition, the rapid release of sodium ions (Figure 7) and potassium ions can be observed over the course of 15 days, caused by the dissolution of the silicate phase present in the composite biomaterial. The increase in silicon concentration is attributed to the loss of SiO_4_^4−^ tetrahedral units from the material surface and the formation of silanol (Si-OH) groups. Overall, the rapid dissolution rate observed can be directly attributed to the high amount of sodium strongly disrupting Si-O-Si connectivity in the bio-glass network, thus reducing its integrity and leading to a higher and more constant dissolution rate [4].

The results show the following concentration variations: calcium concentration decreases over time, while silicon concentration increases due to dissolution of the glass surface over the first 21 days, followed by significant stabilization.

These results are due to the dissolution of the glass in contact with the SBF solution, the formation of a silica gel layer, and finally the formation of a calcium phosphate crystal. This mechanism can be summarized in three successive phases: dissolution of the glass matrix, formation of silica gel, and precipitation of calcium phosphate with an apatitic structure. Based on the results obtained, we can emphasize that this new composite support could be a promising bone substitute due to its bioactivity and dissolution capacity.

### 2.5. Element Distribution on the Surface of Composite Biomaterials

Scanning electron microscopy reveals significant changes in the biomaterials after 37 days immersion in the SBF solution. Typical needle-shaped apatite crystals and large blocky sodium silicate gel formations surrounding small crystals are observed in all composite biomaterials, confirming the formation of interpenetrating matrices where the polymerized gel fills the pores of the composite biomaterial.

**Figure 8:**
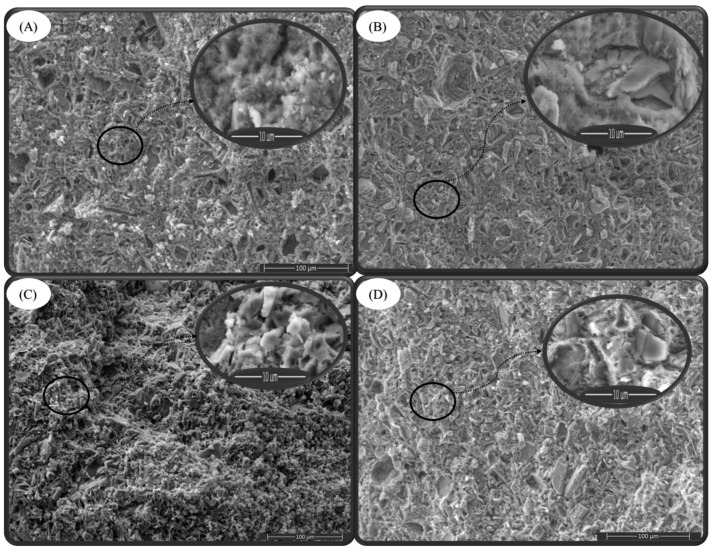
SEM images of composite biomaterials before immersion in SBF (DCPD-Na-M (**A**), DCPD-K-M (**C**)) and after immersion in SBF (37 days) (DCPD-Na-M (**B**), DCPD-K-M (**D**)).

In the case of the DCPD-Na-M composite biomaterial (Figure 8A), an increased porosity is observed compared to the biomaterial before immersion in the SBF solution (Figure 8B). This observation can be attributed to the dissolution of the biocomposite constituents in the SBF solution, including the antibiotic. Similarly, for the DCPD-K-M composite biomaterial (Figure 8C), porosity became greater compared to the biomaterial before immersion in the SBF solution (Figure 8D). This increase in porosity can also be attributed to ionic and molecular exchange with the SBF solution [29].

**Figure 9:**
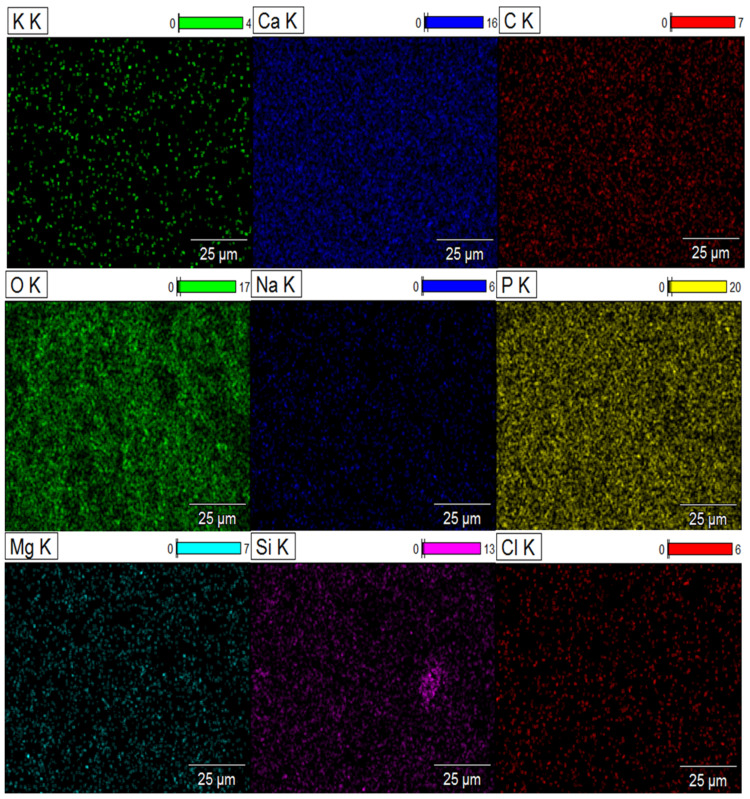
Mapping of DCPD-K-M composite biomaterial after 37 days immersion in SBF solution.

**Figure 10:**
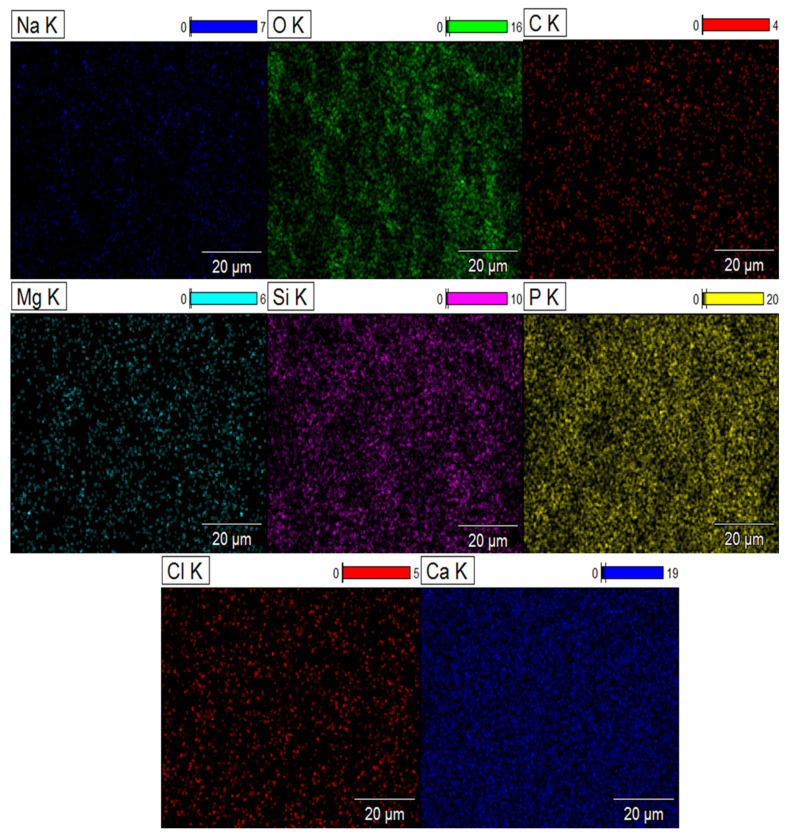
Mapping of DCPD-Na-M composite biomaterial after 37 days immersion in SBF solution.

Scanning electron microscopy thus highlights the structural changes in biomaterials after immersion in the SBF solution. Mapping clearly reveals the distribution of elements on the surface of composite biomaterials after 37 days of immersion in the SBF solution. The signal intensity of each ion is reflected in the intensity of each image. As illustrated in Figure 9 and Figure 10, the elements calcium (Ca), phosphorus (P), sodium (Na), oxygen (O), silicon (Si), potassium (K), chlorine (Cl), carbon (C) and magnesium (Mg) are represented by different colors on the distribution maps. The distribution of the composite biomaterial particles was found to be homogeneous.

The results highlight ionic exchange between the SBF solution and the composites, characterized by the appearance of the elements chlorine (Cl), magnesium (Mg), and sodium (Na) in the DCPD-K-M biocomposite and potassium (K) in the DCPD-Na-M biocomposites.

### 2.6. Toxicity Study

To ensure the suitability of developed biomaterials for applications involving human contact, it is essential to verify that they exhibit no signs of toxicity or adverse health effects. Conducting toxicity tests on these biomaterials is crucial in this regard, as it helps assess any potential harmful or toxic effects they may have on living organisms. These toxicity assays help determine whether the biomaterials can induce adverse reactions such as skin irritation, allergic responses, hormonal disruptions, or other health complications.

In order to assess the toxicity of our composite biomaterial, a series of rigorous experiments were conducted involving three groups (G1, G2, and G3) of albino mice to which different doses of the biomaterial were administered. The doses were meticulously selected within a range of 5 mg/kg (G1), 10 mg/kg (G2), and 15 mg/kg (G3) to allow for precise observation of potential effects over a 15-day period. This duration was deemed adequate to detect any signs of short-term toxicity. The results obtained are promising, as no mortality or significant weight changes were observed in the treated mice during the course of the experiment. This finding is particularly significant as it suggests a favorable tolerance of the composite biomaterial by the mice, thereby reinforcing its potential viability for future applications. Furthermore, the absence of any notable adverse effects, as well as the stability of the mice’s behavior throughout the experiment, also indicate the acceptability of the composite biomaterial by the animals. These observations are crucial for evaluating the safety and suitability of the biomaterial, not only from a toxicological standpoint but also in terms of practical applicability.

### 2.7. Antibacterial Activity

A comparative study of the activity of the DCPD-Na and DCPD-K composite biomaterials in the presence and absence of medication after a 37-day release was conducted on four different bacterial strains: *Listeria monocytogenes*, *Staphylococcus aureus*, *Escherichia coli*, and *Pseudomonas aeruginosa*. Table 2 revealed that both medication-free biomaterials showed no antibacterial activity. However, in the case of the DCPD-Na-M biocomposite, inhibition zones of 10.1, 10.1, 10.5, and 9.5 mm were measured, respectively, against *L*. *monocytogenes*, *S. aureus*, *E. coli*, and *P. aeruginosa*, indicating medication release. Similarly, antibacterial tests of DCPD-K-M showed inhibition zones of 8.5, 8.5, 12.1, and 8.1 mm, respectively, against *L. monocytogenes*, *S. aureus*, *E. coli*, and *P. aeruginosa*. These results suggest that the prolonged release of medication from the composite biomaterials leads to effective antibacterial activity.

This sustained antibiotic release from the composite biomaterials offers potential benefits in medical applications, particularly in the field of bone surgery. Indeed, it may help prevent post-operative infections by providing continuous antibacterial protection over an extended period, thereby reducing the risk of complications and improving clinical outcomes for patients. Unlike antibiotics administered alone, which are rapidly eliminated by the body.

## 3. Materials and Methods

### 3.1. Materials

The reagents used in this study are calcium nitrate tetrahydrate Ca(NO_3_)_2_, 4H_2_O (99%), ammonium hydrogen phosphate (NH_4_)_2_HPO_4_ (99%), high-purity SiO_2_ grade, Davisil Grade 633, NaOH pellets, and KOH pellets. They were all purchased from Sigma-Aldrich, Saint-Quentin-Fallavier, France, and were used without further purification.

### 3.2. Attenuated Total Reflectance Fourier Transform Infrared Spectroscopy (ATR-FTIR)

ATR-FTIR was used to evaluate the formation of composite biomaterials. The spectra were obtained using a FT/IR-4700 spectrometer (JASCO International Ltd., Tokyo, Japan). The spectral range was 400–4000 cm^−1^, and each spectrum was an average of 32 scans at a resolution of 4 cm^−1^. A background check was performed before each analysis.

### 3.3. UV–Visible Spectrophotometry

The analysis of antibiotic concentration in the museum study was carried out by UV–visible spectrophotometry, using a high-performance dual-beam spectrophotometer, the Shimadzu UV-1900 I PC spectrometer manufactured by Shimadzu Duisburg, Germany. This spectrometer covers a spectral range from 190 to 1100 nm.

### 3.4. X-ray Diffraction Analysis (XRD)

To study the crystal structure of the scaffolds and confirm the formation of HAp particles in situ, XRD was performed using the Panalytical X’Pert Pro instrument (Malvern Panalytical GmbH, Kassel, Germany) with a Cu Kα X-ray source at 40 kV and 30 mA, and a scan speed of 2°/min.

### 3.5. Scanning Electron Microscopy (SEM) with Energy-Dispersive X-ray Spectroscopy (EDX)

SEM images were obtained using a Thermo ScientificTM Quattro ESEM (ThermoFisher Scientific, Paisley, UK) at an accelerating voltage of 15 kV. The films were sputter coated with gold before imaging to improve the electron beam conductivity. EDX spectra were acquired using an attached detector on the same instrument.

### 3.6. Inductively Coupled Plasma Atomic Emission Spectrometer (ICP-AES)

ICP analyses were performed using a Horiba Jobin Yvon Ultima 2 ICP-AES spectrometer equipped with a temperature-controlled optical system comprising two back-to-back 4343 tr/mm and 2400 tr/mm gratings covering the spectral range from 120 to 800 nm, with nitrogen scanning. The detection system consists of two photomultipliers.

### 3.7. Zeta Potential

The zeta potential of biomaterial particles was measured using the Anton Paar Litesizer 500 instrument at 25°. Zeta potential values were obtained automatically using the instrument’s software, Kalliope version 2.22.2, which converts the measured electrophoretic mobility into zeta potential in order to evaluate variations in the latter for particles of the materials processed.

### 3.8. Preparation of Antibiotic-Loaded Composite Biomaterials

#### 3.8.1. Synthesis of Dicalcium Phosphate by the Double Decomposition Method

The double decomposition method is often used to obtain a solid precipitate from two clear reactive solutions. It is based on the reaction of two salts dissolved in separate solutions, which exchange ions to form an insoluble precipitate.

In this case, a two-stage synthesis process is used. In the first stage, two solutions, (A) and (B), are prepared and used as reagents. A certain mass of diammonium phosphate is dissolved in distilled water to obtain phosphate solution (A). At the same time, one mass of calcium nitrate is dissolved in one volume of distilled water to obtain calcium solution (B). Both solutions are stirred until they are completely clear. Once the two solutions have been prepared, they are mixed, and the reaction medium is left to stir for 15 min. Then, stop stirring and leave the solution to mature for 2 h. The reaction is as follows:**H_2_O** **(NH_4_)_2_HPO_4_ + Ca(NO_3_)_2_.4H_2_O**
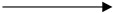
**CaHPO_4_.2H_2_O + 2NH_4_NO_3_**(1)

After ripening, the resulting dicalcium phosphate is filtered and dried overnight at 40 °C.

#### 3.8.2. Synthesis of Sodium Silicate and Potassium Silicate Solutions

Silicate solutions are generally presented as a dispersion of colloidal silica species in an alkaline aqueous solution. In this study, we focused on a concentrated sodium silicate or potassium silicate solution with a low molar ratio (SiO₂/Na₂O = 1 or SiO₂/K₂O = 1) due to the high reactivity of their non-condensed silica species.

For this purpose, a solution of sodium silicate or potassium silicate (Na₂SiO₃, H₂O or K₂SiO₃, H₂O) with a molar ratio of 1 was prepared at 90 °C. Pure NaOH (or pure KOH) pellets were dissolved in deionized water, and then an appropriate amount of commercial silica gel was added. This yielded a liquid glass containing 25% SiO₂ by weight, 25% Na₂O (or 25% K₂O) and 50% water, with a pH of approximately 12. The resulting solution was aged for a short period to ensure the stability of the final state.

#### 3.8.3. Synthesis of Antibiotic-Loaded Composite Biomaterials

The active ingredients are introduced into the initial composition of the dicalcium phosphate/bio-glass solution, undergoing in situ evolution towards the apatite phase, according to the following process:

A solution (A) is prepared by dissolving a precise quantity of drug powder in the corresponding silicate solution. The dicalcium phosphate powder is then gradually added to the solution, paying particular attention to homogenizing the mixture. Solution (A) contains a 1% concentration of the bioactive molecule. The paste is then placed in an airtight container in a humid atmosphere and cured in an oven at 37 °C. The biomaterials synthesized are listed in Table 3.

#### 3.8.4. Study of the Release of Active Ingredients from Engineered Composite Biomaterials

The ability to exchange ions with the immersion medium is crucial in assessing a biomaterial’s ability to promote bonding with living tissue. In vitro tests are therefore carried out to assess this ability. These tests involve immersing samples in a synthetic physiological liquid, SBF (simulated body fluid), whose ionic composition is designed to be similar to that of human blood plasma [30]. SBF is adjusted to a pH of 7.4. Table 4 compares the ionic concentrations of SBF with those of human blood plasma.

**Figure 11:**
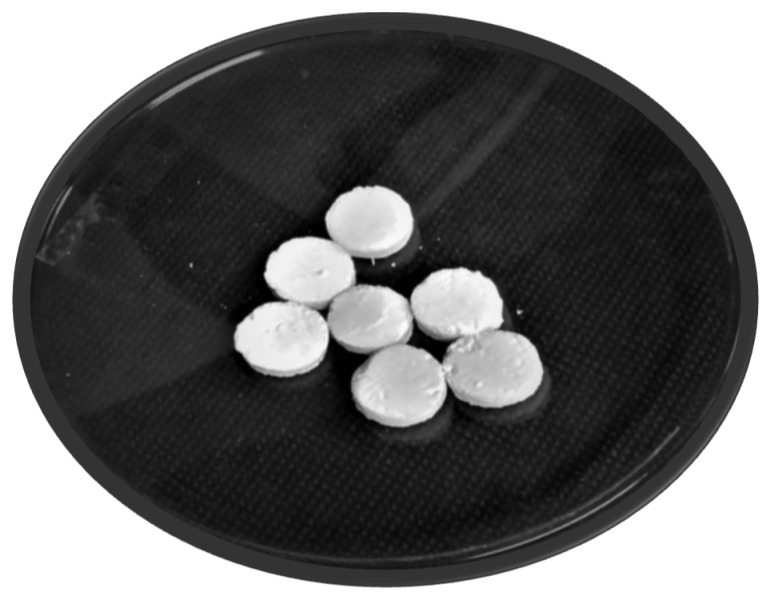
Shaping the sample before immersion in SBF.

The multifunctional composite biomaterials studied are mainly in the form of cylindrical blocks measuring 16.5 mm in diameter by 5 mm in height (Figure 11). This specific configuration was chosen to enable a precise evaluation of the bioactivity kinetics of the various composite biomaterials produced at different points in time.

For in vitro testing, the blocks were placed in hermetically sealed vials and then immersed in 60 mL of SBF. The temperature was maintained at 37 °C under controlled stirring in incubators. The immersion times chosen for the cylinder studies were 5, 7, 14, 21, and 37 days. 

After immersion, the cylinders were immersed in SBF solution. These solutions were then analyzed by UV–visible spectroscopy to monitor the variation in drug concentration over time in the SBF solution. We also monitored ion exchange by ICP-AES and observed possible surface modifications by scanning electron microscopy and possible surface mapping of chemical element distribution in blocks after antibiotic release into the SBF solution.

### 3.9. Test In Vitro

#### 3.9.1. Antibacterial Activity

The in vitro evaluation of antibacterial activity was conducted with two strains of Gram-negative bacteria (*Escherichia coli* ATCC 10536 and *Pseudomonas aeruginosa* ATCC 49189) and two strains of Gram-positive bacteria (*Listeria monocytogenes* ATCC 12117 and *Staphylococcus aureus* ATCC 6538), obtained from the Laboratory of Bioresources, Biotechnologies, Ethnopharmacology, and Health (LBBEH), Faculty of Sciences, Oujda. The bacteria were cultured in Muller-Hinton broth (MHB; BIOKAR, Beauvais, France) [31].

For each bacterial strain, 1 mL of liquid bacterial suspension was added to 9 mL of MHB and incubated at 37 °C for 24 h until the microbial suspension reached the exponential growth phase. Bacterial cultures were adjusted to 0.5 McFarland’s standard to achieve a concentration of 1.5 × 108 CFU/mL.

The antibacterial activity was evaluated using the well method. This involved punching Muller–Hinton agar (MHA) inoculated with the test bacteria (100 µL) to create wells, which were then filled with 60 µL of extract. Negative controls were performed using dimethyl sulfoxide (DMSO), and positive controls were performed using gentamicin.

Cultures were incubated for 18 h at 37 °C after a 30 min pre-diffusion at room temperature [32]. The zone of inhibition around the well was measured in mm using a sliding caliper after incubation.

#### 3.9.2. Toxicity Tests

Swiss albinos were maintained at a temperature of 25 °C with a relative humidity of 45–50% on a 12 h light/dark cycle, with free access to food and water. Prior to the experiments, mice were compared in terms of body weight (between 35 and 30 g). All animals were treated in full compliance with internationally recognized standards for the care and use of laboratory animals, as published by the US National Institutes of Health (NIH Publication No. 85-23, revised 1985).

An initial group of 16 mice was divided into 4 groups of 4 (comprising 2 males and 2 females). The first group served as a control, receiving distilled water. The other three groups were treated with increasing doses of the product. The doses administered were selected on the basis of the maximum volume that could be administered orally. For chemical toxicity studies, doses should be between 5 and 5000 mg/kg.

## 4. Conclusions

In our study, we explored a novel approach to the synthesis of multifunctional composite biomaterials. This consolidation method, based on controlled in situ dissolution and precipitation, enabled the creation of complex biocomposites with remarkable properties. We characterized these biomaterials using a variety of techniques, including infrared spectroscopy, X-ray diffraction, scanning electron microscopy, and zeta potential analysis. These analyses revealed chemical transformations, surface changes, and complex interactions between the composites’ constituent elements. Toxicity tests showed good acceptability. In vitro tests revealed the ability of these biomaterials to exchange ions and active ingredients with a medium simulating body fluid, demonstrating their potential bioactivity. In addition, antibiotic release kinetics were studied, revealing significant differences between composites in terms of antibiotic release and retention. Finally, our experiments demonstrated the antibacterial efficacy of these composites, paving the way for promising applications in the biomedical field. By incorporating the antibiotic into the biomaterial composites, we were able to control its release and prolong its antibacterial activity over time, enabling the development of biocomposites capable of simultaneously playing an osteogenerative role and releasing active ingredients in a continuous and prolonged manner for medical purposes. This research contributes to the advancement of biomaterials by exploring new synthetic routes and demonstrating their potential in the fields of regenerative medicine and controlled drug release. Future studies will further develop these results and explore the practical applications of these innovative biomaterials.

## Figures and Tables

**Table 1:** Zeta potentials of engineered composite biomaterials.

Charged Particle			
Composite Biomaterial	Average Zeta Potential ζ [mV]	Distribution Peak [mV]	Electron Mobility [µm × cm/Vs]
DCPD	−26.6	−24.0	−2.076
DCPD-Na	−23.9	−0.0202	−1.8658
DCPD-Na-M	−19.7	−0.0158	−1.5318
DCPD-K	−24.6	−19.5	−1.9183
DCPD-K-M	−16.5	−0.0125	−1.2847

**Table 2:** Inhibition diameter (mm) of composite biomaterial tested four bacterial strains.

	DCPD-Na	DCPD-K	DCPD-Na-M	DCPD-K-M	Cycloheximide	DMSO
*Escherichia coli*	ND *	ND	10.5 ± 0.1	12.1 ± 0.3	31 ± 0.1	ND
*Pseudomonas aeruginosa*	ND	ND	9.5 ± 0.1	8.1 ± 0.1	21 ± 0.2	ND
*Staphylococcus aureus*	ND	ND	10.1 ± 0.2	8.5 ± 0.1	30.7 ± 0.3	ND
*Listeria monocytogenes*	ND	ND	10.1 ± 0.3	8.5 ± 0.2	30.3 ± 0.2	ND

* Not determined.

**Table 3:** Prepared composite biomaterials.

Composite	SiO_2_/M_2_O	Antibiotic 1%
DCPD-Na	SiO_2_/Na_2_O	0%
DCPD-K	SiO_2_/K_2_O	0%
DCPD-Na-M *	SiO_2_/Na_2_O	1%
DCPD-K-M *	SiO_2_/K_2_O	1%

* M refers to streptomycin sulfate.

**Table 4:** Ionic concentrations of SBF and human blood plasma in mM [30].

Ions (mM)	Na^+^	K^+^	Mg^2+^	Ca^2+^	Cl^−^	HCO^3−^	HPO_4_^2−^	SO_4_^2−^
SBF	142.0	5.0	1.5	2.5	147.8	4.2	1.0	0.5
Plasma	142.0	5.0	1.5	2.5	103.0	27.0	1.0	0.5

## Data Availability

The data presented in this study are available upon request from the corresponding author.

## References

[1] Dornelas J., Dornelas G., Rossi A., Piattelli A., Di Pietro N., Romasco T., Mourão C.F., Alves G.G. (2024). The Incorporation of Zinc into Hydroxyapatite and Its Influence on the Cellular Response to Biomaterials: A Systematic Review. J. Funct. Biomater..

[2] Abdelhamid M.A., Khalifa H.O., Ki M.-R., Pack S.P. (2024). Nanoengineered Silica-Based Biomaterials for Regenerative Medicine. Int. J. Mol. Sci..

[3] Alizadeh-Osgouei M., Li Y., Wen C. (2019). A comprehensive review of biodegradable synthetic polymer-ceramic composites and their manufacture for biomedical applications. Bioact. Mater..

[4] Kargozar S., Ramakrishna S., Mozafari M. (2019). Chemistry of biomaterials: Future prospects. Curr. Opin. Biomed. Eng..

[5] Swetha M., Sahithi K., Moorthi A., Srinivasan N., Ramasamy K., Selvamurugan N. (2010). Biocomposites containing natural polymers and hydroxyapatite for bone tissue engineering. Int. J. Biol. Macromol..

[6] Azzaoui K., Jodeh S., Mejdoubi E., Hammouti B., Taleb M., Ennabety G., Berisha A., Aaddouz M., Youssouf M., Shityakov S. (2023). Synthesis of hydroxyapatite/polyethylene glycol 6000 composites by novel dissolution/precipitation method: Optimization of the adsorption process using a factorial design: DFT and molecular dynamic. BMC Chem..

[7] Magnaterra G. (2020). Additive Manufacturing of Hydroxyapatite Scaffolds for Bone Repair. Ph.D. Thesis.

[8] Pu’ad N.M., Haq R.A., Noh H.M., Abdullah H., Idris M., Lee T. (2020). Synthesis method of hydroxyapatite: A review. Mater. Today Proc..

[9] DileepKumar V., Sridhar M.S., Aramwit P., Krut’ko V.K., Musskaya O.N., Glazov I.E., Reddy N. (2022). A review on the synthesis and properties of hydroxyapatite for biomedical applications. J. Biomater. Sci. Polym. Ed..

[10] El-Ghannam A. (2005). Bone reconstruction: From bioceramics to tissue engineering. Expert Rev. Med. Devices.

[11] Pantulap U., Arango-Ospina M., Boccaccini A.R. (2022). Bioactive glasses incorporating less-common ions to improve biological and physical properties. J. Mater. Sci. Mater. Med..

[12] El Hammari L., Latifi S., Saoiabi S., Azzaoui A., Hammouti B., Chetouani A., Sabbahi R. (2022). Toxic heavy metals removal from river water using a porous phospho-calcic hydroxyapatite. Moroc. J. Chem..

[13] Apelt D., Theiss F., El-Warrak A., Zlinszky K., Bettschart-Wolfisberger R., Bohner M., Matter S., Auer J.A., von Rechenberg B. (2004). In vivo behavior of three different injectable hydraulic calcium phosphate cements. Biomaterials.

[14] Şahin E. (2012). Synthesis and Characterization of Calcium Phosphate Cement Based Macroporous Scaffolds.

[15] Dorozhkin S.V., Kaur G. (2017). Calcium orthophosphate-based bioceramics and its clinical applications. Clinical Applications of Biomaterials.

[16] Nabiyouni M., Brückner T., Zhou H., Gbureck U., Bhaduri S.B. (2018). Magnesium-based bioceramics in orthopedic applications. Acta Biomater..

[17] Atila D., Dalgic A.D., Krzemińska A., Pietrasik J., Gendaszewska-Darmach E., Bociaga D., Lipinska M., Laoutid F., Passion J., Kumaravel V. (2024). Injectable Liposome-Loaded Hydrogel Formulations with Controlled Release of Curcumin and α-Tocopherol for Dental Tissue Engineering. Adv. Healthc. Mater..

[18] Mansour A., Romani M., Acharya A.B., Rahman B., Verron E., Badran Z. (2023). Drug delivery systems in regenerative medicine: An updated review. Pharmaceutics.

[19] Gao P., Nie X., Zou M., Shi Y., Cheng G. (2011). Recent advances in materials for extended-release antibiotic delivery system. J. Antibiot..

[20] Chindamo G., Sapino S., Peira E., Chirio D., Gonzalez M.C., Gallarate M. (2020). Bone diseases: Current approach and future perspectives in drug delivery systems for bone targeted therapeutics. Nanomaterials.

[21] Liang W., Zhou C., Jin S., Fu L., Zhang H., Huang X., Long H., Ming W., Zhao J. (2023). An update on the advances in the field of nanostructured drug delivery systems for a variety of orthopedic applications. Drug Deliv..

[22] Aaddouz M., Azzaoui K., Akartasse N., Mejdoubi E., Hammouti B., Taleb M., Sabbahi R., Alshahateet S. (2023). Removal of methylene blue from aqueous solution by adsorption onto hydroxyapatite nanoparticles. J. Mol. Struct..

[23] Azzaoui K., Aaddouz M., Akartasse N., Mejdoubi E., Jodeh S., Hammouti B., Taleb M., ES-Sehli S., Berisha A., Rhazi L. (2023). Synthesis of β-Tricalcium Phosphate/PEG 6000 Composite by Novel Dissolution/Precipitation Method: Optimization of the Adsorption Process Using a Factorial Design—DFT and Molecular Dynamic. Arab. J. Sci. Eng..

[24] Degli Esposti L., Adamiano A., Siliqi D., Giannini C., Iafisco M. (2021). The effect of chemical structure of carboxylate molecules on hydroxyapatite nanoparticles. A structural and morphological study. Bioact. Mater..

[25] Walsh D., Kingston J.L., Heywood B.R., Mann S. (1993). Influence of monosaccharides and related molecules on the morphology of hydroxyapatite. J. Cryst. Growth.

[26] Aaddouz M., Azzaoui K., Sabbahi R., Youssoufi M.H., Yahyaoui M.I., Asehraou A., El Miz M., Hammouti B., Shityakov S., Siaj M. (2023). Cheminformatics-based design and synthesis of hydroxyapatite/collagen nanocomposites for biomedical applications. Polymers.

[27] Khin S.Y., Soe H.M.S.H., Chansriniyom C., Pornputtapong N., Asasutjarit R., Loftsson T., Jansook P. (2022). Development of fenofibrate/randomly methylated β-cyclodextrin-loaded Eudragit® RL 100 nanoparticles for ocular delivery. Molecules.

[28] Cerchiara T., Abruzzo A., Di Cagno M., Bigucci F., Bauer-Brandl A., Parolin C., Vitali B., Gallucci M., Luppi B. (2015). Chitosan based micro-and nanoparticles for colon-targeted delivery of vancomycin prepared by alternative processing methods. Eur. J. Pharm. Biopharm..

[29] Đošić M.S., Mitrić M., Mišković-Stanković V.B. (2015). The porosity and roughness of electrodeposited calcium phosphate coatings in simulated body fluid. J. Serbian Chem. Soc..

[30] Kokubo T., Kushitani H., Sakka S., Kitsugi T., Yamamuro T. (1990). Solutions able to reproduce in vivo surface-structure changes in bioactive glass-ceramic A-W3. J. Biomed. Mater. Res..

[31] Celiktas O.Y., Kocabas E.H., Bedir E., Sukan F.V., Ozek T., Baser K. (2007). Antimicrobial activities of methanol extracts and essential oils of Rosmarinus officinalis, depending on location and seasonal variations. Food Chem..

[32] Pundir C.S., Rawal R. (2013). Determination of sulfite with emphasis on biosensing methods: A review. Anal. Bioanal. Chem..

